# Utilization of Dental Care and Oral Health Outcomes in the United States: Results from the National Health and Nutrition Examination Survey (2017–2020)

**DOI:** 10.3290/j.ohpd.b5680746

**Published:** 2024-08-13

**Authors:** Hannah R. Archer, Nicky (Huan) Li, Erinne Kennedy, Muath A. Aldosari

**Affiliations:** a MPH, MAEd, Student, University of North Carolina, Adams School of Dentistry, Chapel Hill, NC, USA. Contribution to the study: Methods, Data Interpretation, First Author/Primary Writer.; b Kerr Corporation, USA, Methods, Data Analysis, Data Interpretation.; c DMD, MPH, MMScAssistant Dean for Integrated Learning, Data Interpretation, Design and Draft of Manuscript.; d Assistant Professor, Periodontics and Community Dentistry, King Saud University College of Dentistry, Riyadh, Saudi Arabi, Methods, Data Analysis, Data Interpretation, Study Design.

**Keywords:** dental appointments, dental attendance, dental caries, oral health disparities, routine care, dental appointments

## Abstract

**Purpose:**

This analysis aims to evaluate the association between the time since and reason for a patient’s last dental appointment across clinical oral health outcomes.

**Materials and Methods:**

We used data from the 2017–2020 National Health and Nutrition Examination Survey (NHANES), a cross-sectional, nationally-representative survey of noninstitutionalized US adults. The predictors were the time since and the reason for the last dental appointment (routine vs. urgent). We examined the presence and number of missing teeth and teeth with untreated coronal and root caries. Multivariable regression models with interaction were used to assess the association between the time since the last dental appointment and clinical oral health outcomes among routine and urgent users separately.

**Results:**

Two-thirds of the US population had a dental appointment within a year, while nearly 44 million individuals did not visit a dentist for the last three years. The odds of having teeth with untreated coronal or root caries increased with the length of time since the last appointment, and urgent users had worse dental outcomes compared to routine users. Compared to those who had a dental appointment within a year, individuals who had their last dental appointment more than 3 years ago had 2.94 times the average number of teeth with untreated caries among routine users (95%CI=2.39, 3.62) and 1.60 times the average among urgent users (95%CI=1.05, 2.43).

**Conclusions:**

Recent, routine dental appointments are associated with improved oral health outcomes. The outcomes reiterate how social determinants of health impact access to oral health care and oral health outcomes.

While policy makers, healthcare professionals, and health systems have been working since 2000 to “put the mouth back in the body,” patients still report challenges with receiving consistent dental care.^9,13^ Dental caries affects 193.5 million U.S. adults and one out of 10 children, which is a result of a variety of environmental, behavioral, social, and biological factors.^1,8^ It is one of the most preventable chronic diseases.

Social determinants of health (SDOH) contribute significantly to an individual’s oral health status. Patients who have a low income, are uninsured or underinsured, belong to underrepresented minority groups, or live in rural areas are more likely to experience poor oral health. SDOH may create barriers in an individual’s ability to find, access, or afford quality oral health care.^12^ As a result, socioeconomic inequality creates disparities in oral health outcomes among different populations.^10,14^

Dentists create preventive re-care plans with appointments for regular evaluation based on patient risk factors. The evaluation interval can range from 3 months for high-risk patients to 1 year for healthy individuals.^6^ More frequent appointments allow the oral health care team to assess risk factors, detect early disease indicators, and provide prevention at the earliest signs of disease.^7,11^ Even healthy patients may benefit from frequent oral health care visits to sustain population level health.

The number and consistency of dental appointments per year influence oral health outcomes, but how it influences it is not clear.^16^ Previous research examined the differences in oral health outcomes between adults with routine dental appointments and adults with appointments in response to a specific problem or urgency. The results suggest conflicting ideas such that more frequent dental appointments coincide with the disadvantage of more frequent treatment, such as an increase in fillings, and thus a greater disease experience. However, more frequent dental appointments also coincide with the advantage of more restored teeth and less active or untreated decay.^12^ More recent research finds similar advantages to more frequent dental appointments including less overall tooth decay and better oral health outcomes. However, these studies examined the relationship using small, non-representative samples. In addition, none reported the magnitude of difference in oral diseases in relation to the length of time since the patient’s last dental appointment, or the difference between individuals who had a dental appointment for routine care compared to those who had an urgent appointment.^3,18^

This study uses the nationally representative data from the National Health and Nutrition Examination Survey (NHANES) 2017–March 2020 pre-pandemic cycle to examine the elapsed time between the last dental appointment to current oral conditions and to determine whether the last dental appointment was routine or urgent. Our primary aim is to assess the association between the time since the patient’s last dental appointment (primary exposure) and oral health outcomes. The outcomes measured here include the presence and number of coronal caries, the presence of root caries, and the presence and number of missing teeth. The secondary aims are to investigate the socioeconomic inequalities in access to dental care and to explore oral health outcomes from routine versus urgent dental appointments.

An a priori hypothesis is that those who had recent dental appointments (<1 year) for routine care will have better oral health outcomes (fewer number of teeth with coronal caries, lower probability of having root caries, and fewer missing teeth) compared to patients who had dental appointments more than a year ago or for urgent care. Lack of oral health access and socioeconomic inequalities are associated with increased untreated dental conditions (i.e. coronal caries, root caries, and more missing teeth).

## MATERIALS AND METHODS

### Study Design and Population

NHANES is a cross-sectional survey of non-institutionalized US civilians to collect data through a combination of laboratory assessments, self-reported questionnaires, and clinical exams. It is conducted bi-annually. However, the COVID-19 pandemic prevented field operations in March 2020, which resulted in the incomplete data collection for the 2019-2020 cycle, making the collected data not nationally representative. To address this issue, the data collected from 2019 until March 2020 were combined with data from the NHANES 2017-2018 cycle to create a nationally representative sample of NHANES 2017–March 2020 pre-pandemic data.^15^

The unweighted response rate of the examined sample was 47% for the 2017–March 2020 cycle. We included all participants one year or older who ever had a dental visit and completed the dental exam with at least one natural tooth, exclusive of third molars, and answered the questions about their last dental appointment. The total sample size included 12,200 participants, and all participants provided written informed consent prior to study participation. The study was approved by the ethical review boards of the National Center for Health Statistics (approval protocol numbers: 2011–17 and 2018–01).^4^ Informed consent was obtained during the NHANES survey. Survey participants were assured that no information could be linked back to them or any other individual during the informed consent process. Since our study is a secondary analysis of publicly available data, no additional ethical approval was necessary.

### Clinical Assessment of Oral Diseases

Trained and calibrated dental professionals conducted all the clinical examinations to assess oral health status. We described teeth with untreated coronal caries as any dental cavity in the crown of a tooth that was both active and untreated, excluding third molars. For adults aged 20 years or older, we defined untreated root caries as any carious lesion located below the cementoenamel junction and above the gingival margin of teeth with gum recession, excluding third molars. We categorized missing teeth as teeth that had been lost due to caries or periodontal disease. Additionally, we determined the number of teeth that had coronal caries and the number of missing teeth.

### Utilization of Dental Care and Demographic Factors

The primary predictor was the time since the last dental appointment. Participants were asked, “When did you last visit a dentist?” We categorized participants into three groups: if they had a dental appointment within the previous year; if they had one more than a year before, but within three years; and if their last appointment was more than 3 years ago.

We further categorized participants based on whether their appointments had been routine or urgent. Using their answers to the question “What was the main reason you last visited the dentist?”, participants were considered routine dental care attendees if they answered “Went in on own for check-up, examination, or cleaning”; “Was called in by the dentist for check-up, examination, or cleaning”; or “Went for treatment of a condition that dentist discovered at earlier checkup or examination.” Urgent attendees were identified if they answered “Something was wrong, bothering or hurting me.”

Based on a theoretical framework presented in the directed acyclic graph (Appendix 1), we took into account the following sociodemographic confounders: age, gender, race/ethnicity, family income based on federal poverty level, and education level. The age variable was divided into seven groups (1-5, 6-11, 12-19, 20-34, 35-49, 50-64, 65+). Gender was either male or female. The race/ethnicity variable was divided into five groups, including Non-Hispanic Asian, non-Hispanic Black, non-Hispanic White, Mexican American/Hispanic, and Other, which included multi-racial groups. Family income was divided into four groups based on the ratio of family income to the federal poverty level (FPL). Finally, education level was reported based on the highest grade of school completed or the highest degree the participant received, then divided into five groups: younger than 20 years old (education not reported), less than high school, completed high school/GED, some college or AA degree, and college graduate or above.

**Appendix 1:**
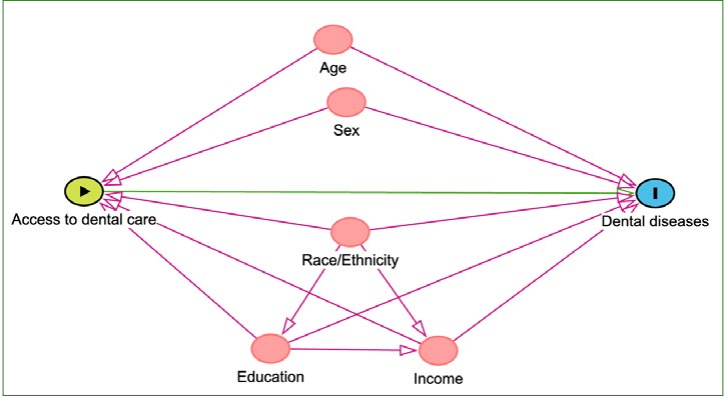
Directed acyclic graph (DAG) of the causal relationship between access of dental services, dental diseases, and the confounding factors.

### Statistical Analysis Plan

We described first the demographic distribution of our study population. We used Pearson’s chi-square test to assess the distribution of these characteristics by the time since their last dental appointment. We reported the prevalence of teeth with untreated coronal caries, teeth with root caries, whether any tooth was missing, the mean number of teeth with untreated coronal caries, and the mean number of missing teeth. National weighted estimates were reported with the corresponding 95% confidence intervals (95% CI) and stratified by the reason of dental appointments. Taylor linearization methods were used in the survey procedures for standard error estimations with the publicly provided masked variance pseudoprimary and masked variance pseudostratum sampling units.

Logistic regressions were used for the binary dental outcomes: presence of untreated coronal caries, presence of untreated root caries, and presence of missing teeth. Poisson regressions were used to assess the mean ratio for the count outcomes: number of teeth with untreated coronal caries and number of missing teeth. Simple logistic/Poisson regressions were run first to assess the crude estimates of the oral health outcomes by the time since the last dental appointment, stratified by the reason for the appointment (routine or urgent). Then, we adjusted for demographic characteristics in the final multiple regression models with the interaction between the time since the last dental appointment and the reason for the appointment. Alpha was set at 0.05, and all analyses were carried out using Stata 17.0 (StataCorp; College Station, TX, USA).

## RESULTS

The datasets generated and analyzed during the current study are available in the Centers for Disease Control and Prevention National Health and Nutrition Examination Survey (NHANES) repository, https://www.cdc.gov/nchs/nhanes/index.htm.

Nearly two-thirds of the US population had an appointment with a dental professional within a year of the survey (Table 1). Sociodemographic factors were associated with the recency of the last dental visit. Recency was the highest among children ≤ 5 years old (94.4%), females (70.1%), non-Hispanic White individuals (60.2%). In addition, having a dental appointment within a year increased as income and education increased. However, almost 53 million individuals did not have a dental appointment for more than three years. This was highest among young adults 20–34-year-old (22.3%), non-Hispanic Black individuals (18.7%), and other racial minorities (19.3%).

**Table 1:** Demographics and prevalence of dental utilization among participants who ever had a dental visit and have completed the dental examination in the National Health and Nutrition and Examination Survey, 2017-March 2020

	Overall number n (%)^a^	Within a year		More than 1 year ago, but not more than 3 years ago		More than 3 years ago		p-value^b^
		%	Weighted U.S. population N (in thousands)	%	Weighted U.S. population N (in thousands)	%	Weighted U.S. population N (in thousands)	
Overall	12,200 (100)	67. 0	195,289	18.1	52,677	14.9	43,505	-
Age								
≤5	986 (4.4)	94.4	11,987	5.6	713	0.0	0	<0.01
6-11	1,704 (8.2)	90.0	21,416	9.4	2,227	0.7	156	
12-19	1,757 (10.9)	83.6	26,649	13.3	4,237	3.1	975	
20-34	1,802 (21.3)	52.0	32,216	25.8	15,977	22.3	13,790	
35-49	1,829 (19.1)	59.5	33,059	22.5	12,492	18.0	10,011	
50-64	2,186 (20.3)	65.2	38,662	17.7	10,474	17.1	10,136	
65+	1,936 (15.9)	67.6	31,2976	14.2	6,553	18.2	8,438	
Gender								
Male	5,996 (48.7)	63.8	90,501	18.9	26,809	17.3	24,570	<0.01
Female	6,204 (51.3)	70.1	104,788	17.3	25,868	12.7	18,935	
Race/Ethnicity								
Non-Hispanic White	4,073 (60.2)	69.3	121,573	16.2	28,337	14.5	25,462	<0.01
Mexican American/ Other Hispanic	2,765 (17.8)	64.5	33,490	21.7	11,145	14.1	7,322	
Non-Hispanic Black	3,294 (11.9)	59.7	20,679	21.5	7,512	18.7	6,472	
Non-Hispanic Asian	1,302 (5.5)	70.2	11,338	19.4	3,142	10.4	1,682	
Other, including multi-racial	766 (4.6)	61.6	8,209	19.1	2,541	19.3	2,569	
Education								
Younger than 20 years old (education not reported)	4,455 (23.5)	87.8	60,120	10.5	7,196	1.7	1,154	<0.01
Less than high school	1,371 (7.8)	42.0	9,569	27.0	6,149	31.1	7,077	
High school graduate	1,840 (20.3)	51.6	30,486	22.5	13,275	26.0	15,355	
Some college/AA	2,574 (23.6)	58.7	40,413	22.5	15,473	18.9	13,021	
College graduate or above	1.960 (24.8)	75.8	54,700	14.7	10,583	9.6	6,898	
Income								
<100% FPL^c^	2,454 (13.0)	57.8	21,902	21.0	7,950	21.2	8,052	<0.01
100-199% FPL	2,839 (17.7)	56.6	29,110	22.2	11,439	21.2	10,893	
200-399% FPL	2,758 (25.2)	64.6	47,455	18.8	13,813	16.7	12,246	
>400% FPL	4,149 (44.1)	75.3	96,822	15.1	19,475	9.6	12,314	

^a^The sample counts were unweighted while percentages are weighted to account for complex survey design.

For individuals who reported their last dental appointment was for routine care (Table 2), teeth with untreated coronal caries were present in one out of four individuals who reported their last dental appointment more than three years ago, with an average of 0.70 teeth affected by caries (95%CI=0.52, 0.89). In contrast, teeth with untreated coronal caries were present among only 8.4% of those who had a dental appointment within the year, with an average of 0.18 teeth affected by caries (95%CI=0.15, 0.20). Similarly, teeth with root caries were present among 9.7% of those who had their last dental appointment more than three years ago (95%CI=7.5, 12.0), while only 3.6% who had a dental appointment within last year (95%CI=2.6, 4.5). Almost one-third of all individuals who reported their last dental appointment as routine care had at least one tooth missing due to dental diseases. The highest presence of missing teeth (33.1%) was among those who had their last dental appointment in the previous 1 to 3 years (95%CI=29.0, 37.2). As for those who reported their last appointment as urgent, both the prevalence and average number of dental diseases were higher among all groups, with the highest presence of missing teeth (74.7%) being among those who had their last dental appointment more than 3 years ago (95%CI= 71.0, 78.5).

**Table 2:** Oral health outcomes by type and time since last dental visit among participants who ever had a dental visit and have completed the dental examination in the National Health and Nutrition and Examination Survey, 2017-March 2020

	Coronal caries (N = 11,666)		Root caries (N = 7,288)^a^	Missing teeth (N = 11,764)	
	Presence of untreated coronal caries % (95% CI)	Mean number of teeth withuntreated coronal caries mean (95% CI)	Presence of untreated root caries % (95% CI)	Presence of missing teeth % (95% CI)	Mean number of missing teeth mean (95% CI)
Overall population	16.9 (14.5, 19.3)	0.43 (0.36, 0.50)	10.1 (8.2, 11.9)	38.0 (35.7, 40.4)	2.17 (1.95, 2.40)
Routine visitors					
Visit less than 1 year ago	8.4 (6.9, 9.9)	0.18 (0.15, 0.20)	3.6 (2.6, 4.5)	30.7 (28.4, 33.1)	1.51 (1.33, 1.69)
Visit more than 1 year ago, but not more than 3 years ago	19.9 (16.2, 23.5)	0.47 (0.34, 0.61)	9.0 (5.4, 12.7)	33.1 (29.0, 37.2)	1.84 (1.56, 2.12)
Visit more than 3 years ago	27.4 (22.4, 32.4)	0.70 (0.52, 0.89)	9.7 (7.5, 12.0)	27.0 (23.0, 30.9)	1.80 (1.35, 2.26)
Urgent visitors					
Visit less than 1 year ago	28.8 (24.1, 33.5)	0.80 (0.65 0.96)	21.1 (16.6, 25.6)	64.7 (59.7, 69.7)	4.02 (3.39, 4.65)
Visit more than 1 year ago, but not more than 3 years ago	40.0 (34.5, 45.5)	1.24 (1.03 1.45)	24.9 (19.1, 30.8)	68.7 (65.1, 72.3)	4.45 (3.80, 5.11)
Visit more than 3 years ago	47.2 (38.2, 56.3)	1.37 (0.94 1.79)	31.0 (19.4, 42.7)	74.7 (71.0, 78.5)	5.56 (4.63, 6.49)

95%CI: 95% confidence interval.

^a^Only 20+ year old adults were assessed for root caries-

**Fig 1:**
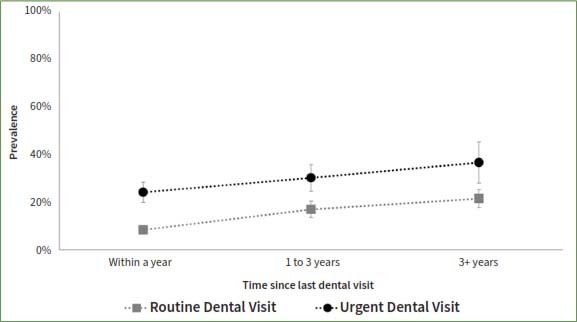
Presence of teeth with untreated coronal caries by reason for and time since the last dental visit among participants who ever had a dental visit and have completed the dental examination in the National Health and Nutrition and Examination Survey, NHANES, 2017-2020.

**Fig 2:**
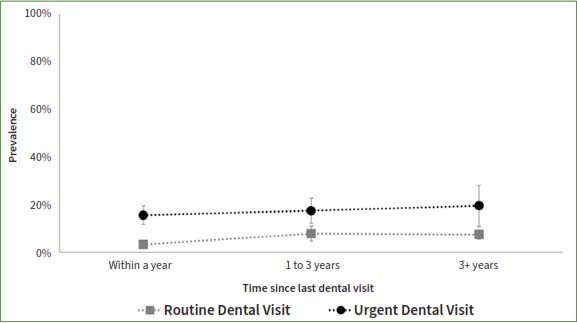
Presence of teeth with untreated root caries by reason of and the time since the last dental visit among 20+ participants who ever had a dental visit and have completed the dental examination in the National Health and Nutrition and Examination Survey, NHANES, 2017-2020.

**Fig 3:**
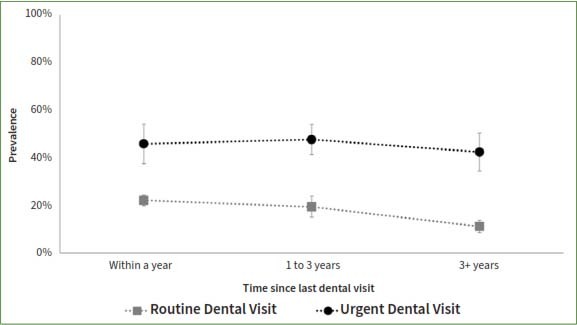
Presence of missing teeth by reason of and the time since the last dental visit among participants who ever had a dental visit and have completed the dental examination in the National Health and Nutrition and Examination Survey, NHANES, 2017-2020.

The study compared patients who reported a dental appointment within a year time frame such that a longer time since the last routine appointment was associated with higher odds of having teeth with untreated coronal or root caries, even after adjusting for socio-demographic factors (Table 3, Figs 1 and 2). In contrast, a longer time since the last dental appointment was associated with lower odds of missing teeth. The adjusted odds of having teeth missing among those who had their last dental appointment for routine care more than 3 years ago was 0.44 times that of those who had a routine appointment within a year (95%CI= 0.33, 0.59). The odds of missing teeth if the last dental appointment was more than 3 years ago was 0.87 times that of those who had an urgent dental appointment within the year (95%CI=0.58, 1.31) (Fig 3).

**Table 3:** Associations between utilization of dental visit and oral health outcomes among participants who ever had a dental visit and have completed the dental examination in the National Health and Nutrition and Examination Survey, 2017-March 2020.

	Presence of untreated coronal caries		Presence of root caries^b^		Presence of missing teeth	
	Crude OR (95%CI)	Adjusted OR^a^ (95%CI)	Crude OR (95%CI)	Adjusted OR^a^ (95%CI)	Crude OR (95%CI)	Adjusted OR^a^ (95%CI)
Routine visitors						
Visit less than 1 year ago	Ref	Ref	Ref	Ref	Ref	Ref
Visit more than 1 year ago, but not more than 3 years ago	2.70 (2.14, 3.40)	2.23 (1.72, 2.88)	2.70 (1.68, 4.33)	2.51 (1.55, 4.05)	1.12 (0.93, 1.34)	0.85 (0.65, 1.11)
Visit more than 3 years ago	4.11 (3.35, 5.03)	2.99 (2.40, 3.71)	2.92 (1.97, 4.31)	2.37 (1.53, 3.67)	0.83 (0.69, 1.00)	0.44 (0.33, 0.58)
Urgent visitors						
Visit less than 1 year ago	Ref	Ref	Ref	Ref	Ref	Ref
Visit more than 1 year ago, but not more than 3 years ago	1.65 (1.22, 2.22)	1.36 (1.00, 1.85)	1.24 (0.87, 1.77)	1.15 (0.79, 1.66)	1.20 (0.93, 1.54)	1.08 (0.71, 1.62)
Visit more than 3 years ago	2.22 (1.45, 3.40)	1.81 (1.19, 2.76)	1.68 (1.02, 2.78)	1.32 (0.81, 2.14)	1.61 (1.21, 2.16)	0.87 (0.58, 1.31)

^a^The model was adjusted for age, sex, race, education, and income.

^b^Only 20+ year old adults were assessed for root caries. 95%CI: 95% confidence interval.

The increase in the average number of untreated coronal caries increased by 2.33 fold if the last dental appointment was in the previous 1-3 years (95%CI=1.67, 3.24) and 2.94 fold if it had been more than three years (95%CI=2.39, 3.62) compared to individuals who had their last dental appointment within a year for routine care, after adjusting for socio-economic confounders (Appendices 2 and 3). In addition to lower odds of having any missing teeth, those with dental appointments for routine care more than three years prior had 0.67 times the adjusted average number of missing teeth compared to those that had dental appointments within the previous year for routine care (95%CI=0.52, 0.86) (Appendix 4).

**Appendix 2:** Associations between utilization of dental visit and average number of teeth with untreated coronal caries and missing teeth among participants who have completed the dental examination in the National Health and Nutrition and Examination Survey, 2017-March 2020.

	Mean number of teeth with untreated coronal caries		Mean number of missing teeth	
	Crude Mean Ratio (95%CI)	Adjusted Mean Ratio (95%CI)	Crude Mean Ratio (95%CI)	Adjusted Mean Ratio^a^ (95%CI)
Routine Visitors				
Visit-less than 1 year ago	Ref	Ref	Ref	Ref
Visit- more than 1 year ago, but not more than 3 years ago	2.69 (1.94, 3.73)	2.33 (1.67, 3.24)	1.22 (1.05, 1.41)	0.95 (0.80, 1.13)
Visit- more than 3 years ago	3.99 (3.27, 4.87)	2.94 (2.39, 3.62)	1.19 (0.93, 1.51)	0.67 (0.52, 0.86)
Urgent Visitors				
Visit-less than 1 year ago	Ref	Ref	Ref	Ref
Visit- more than 1 year ago, but not more than 3 years ago	1.54 (1.21, 1.98)	1.29 (0.98, 1.71)	1.11 (0.88, 1.39)	1.04 (0.80, 1.36)
Visit- more than 3 years ago or never have been	1.70 (1.16, 2.50)	1.60 (1.05, 2.43)	1.38 (1.12, 1.71)	0.87 (0.70, 1.10)

^a^The model was adjusted for age, sex, race, education, and income. 95%CI: 95% confidence interval.

**Appendix 3:**
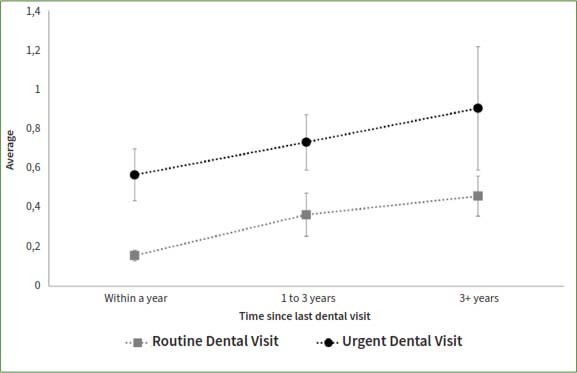
Average number of teeth with untreated coronal caries by reason of and the time since the last dental visit among participants who ever had a dental visit and have completed the dental examination in the National Health and Nutrition and Examination Survey, NHANES, 2017-2020.

**Appendix 4:**
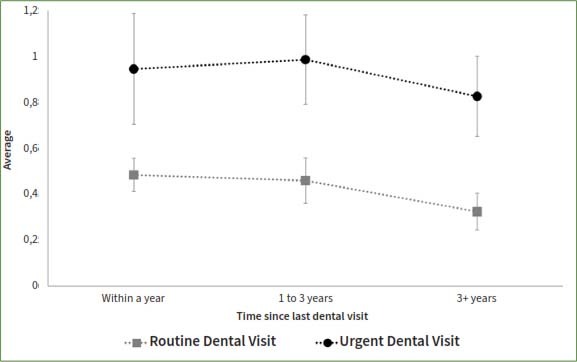
Average number of missing teeth by reason of and the time since the last dental visit among participants who ever had a dental visit and have completed the dental examination in the National Health and Nutrition and Examination Survey, NHANES, 2017-2020.

## DISCUSSION

Previous research suggests the frequency between dental appointments influences health outcomes, but the specific impact on oral health outcomes is yet to be determined.^17^ Our study uses nationally representative data to address current gaps in the literature. This data allows us to explore the association between the self-reported frequency of dental appointments and a clinical report of oral health status. In this study, increased time elapsed since the last dental appointment was associated with a higher presence of untreated caries. Similarly, the longer the time since the last dental appointment, the great number of missing teeth and teeth with untreated coronal caries are observed.

The findings suggest that regular routine dental appointments support improved oral health, and the analysis further depicts how SDOH may impact the frequency between dental appointments.^19^ Mexican American/Other Hispanic populations report the lowest percentage of frequency between dental appointments compared to Non-Hispanic Whites. Less high school education is associated with a lower percentage of dental appointments, with the lowest frequency among populations with less than a high school education. Lower levels of income mirror the same trend such that individuals below 100% FPL report the lowest percentage of dental appointments. In a systematic review by Northridge et al, over twenty articles were assembled to assess interventions that address oral health care disparities. The results of the review indicate a greater likelihood of poor dental health for individuals who are low-income and/or members of racial/ethnic minorities compared to populations with better access to oral health care.^14^ The results from this review align closely with our findings, reiterating the impact of social determinants on the frequency of dental appointments and subsequent oral health outcomes.

The findings expand on previous studies that examine how the type of dental appointments – routine versus urgent – impacts oral health outcomes. Patients that had dental appointments more than three years prior had fewer missing teeth than those who had dental appointments less than three years prior. Similarly, those that had dental appointments more than one year ago had fewer missing teeth than those who had dental appointments less than one year ago. This lower presence of missing teeth among patients with more time since the last dental appointment could be explained by a greater disease experience among patients that have more frequent dental appointments.^17^ Additionally, if the patients had not accessed care for a longer period of time, it is likely they were unable to receive an extraction even if the treatment was needed, which would explain why teeth were retained due to lack of access to care, not lack of disease. This possible explanation is supported by our finding that, regardless of the reason for the dental appointment, those who had dental appointments more than a year ago had more presence of untreated coronal and root caries. In this sense, less time between dental appointments likely results in less untreated caries due to more frequent treatment and/or use of more prevention-based care.^16-18^

Previous research provides conflicting results regarding the relationship between the frequency of dental appointments and the impact this has on oral health outcomes. Sheiham et al^17^ in 1985 reported more frequent dental appointments resulted in a lower rate of tooth loss and fewer teeth with active decay, yet a higher average number of fillings. This article demonstrated the advantages of frequent dental appointments such that patients had more functioning and/or restored teeth. However, Sheiham et al^17^ also reported that increased appointments came at a disadvantage of a higher disease experience, maybe due to over-treatment, indicating frequent dental appointments maintain oral function but do not prevent future disease. Our results are similar to Sheiham et al,^17^ as more frequent dental appointments resulted in less teeth with untreated caries. However, our results differed in the potential association between the recency of dental appointments and the number of missing teeth.

NHANES data provides a relatively large sample size and a rigorous study design. Therefore, the data analyzed in our study are nationally representative and can be generalized to non-institutionalized US civilians. The oral health outcomes are also assessed clinically, which furthers the strength and validity of this study. However, it is important to note that as a cross-sectional study, NHANES is restricted to association evaluations, rather than causality. Dental care utilization examined in our analysis was only the last dental appointment, rather than the number of appointments within a specific period of time. There are also potential limitations with reporting bias, as the timing and reason for the last of dental appointment was self-reported by participants. NHANES also presents limitations in the types of clinical measurements that the survey reports. In the case of our study, the ability to measure the number of teeth with root caries was limited, as NHANES clinically measures root caries at the mouth level such that an individual either had “any root caries” or “no root caries.” Finally, there may be unknown variables affecting the frequency of dental appointments, such as a patient’s risk of dental caries, salivary markers, and oral hygiene practice.^5,17,18^ However, the analysis controlled for potential confounding factors including age, gender, race, education, and income to strengthen the internal validity of our estimates.

## CONCLUSION

There is a significant need for more accessible dental services, particularly for populations more likely to face additional barriers to accessing oral health care.^2,12^ While no direct relationship was found between the frequency of dental appointments and oral health outcomes, our results indicate that consistent and frequent routine dental appointments can have a positive impact on oral health outcomes. Through our findings, we recommend that individuals visit the dentist for frequent, routine care to reduce urgent visits and/or negative oral health outcomes. One potential hurdle to obtaining consistent and frequent dental care is the cost associated with such care.^2^ As such, our findings support the implementation of an insurance policy that covers annual dental preventive appointments, which would save costs and reduce dental-related emergency appointments. Our results also indicate a call to action for clinicians and insurance providers alike to ensure that dental appointments consist primarily of prevention-focused care. Populations that face greater obstacles to access dental care, in particular, can benefit from more frequent and prevention-focused dental care.^14,20^
